# Hierarchical Interfacial Construction by Grafting Cellulose Nanocrystals onto Carbon Fiber for Improving the Mechanical Performance of Epoxy Composites

**DOI:** 10.3390/nano14181537

**Published:** 2024-09-22

**Authors:** Yanjiao Ma, Wei Zhao, Jun Xiong, Wei Zhang, Mingfeng Dai, Yifan Guo, Ying Li, Ling Long, Zuowan Zhou

**Affiliations:** 1 Chengdu Aircraft Industry Group Co., Ltd., Chengdu 610073, China; mayj009@avic.com (Y.M.); zhaow119@avic.com (W.Z.); xiongj004@avic.com (J.X.); 2 School of Chemistry, Key Laboratory of Advanced Technologies of Materials (Ministry of Education), Southwest Jiaotong University, Chengdu 610031, China; zw19102663364@163.com (W.Z.); 18384244170@163.com (M.D.); 3 School of Aeronautical Equipment Manufacturing Industry, Chengdu Aeronautic Polytechnic, Chengdu 610100, China; sclongling@163.com; 4 School of Mechanical Engineering, Chengdu University, Chengdu 610106, China; liying@cdu.edu.cn

**Keywords:** carbon fiber, epoxy composites, cellulose nanocrystal, mechanical properties

## Abstract

Carbon fiber-reinforced composites have been widely used in the aerospace industry because of their superior comprehensive performance, including high strength, low density, fatigue resistance, long service life, etc. The interface between the fiber reinforcement and the matrix is one of the key factors that determines the performance of the composites. The construction of covalent bonding connections between the components has proven to be an effective strategy for improving the interfacial bonding strength but always reduces the toughness. In this work, dual silane coupling agents are applied to covalently connect cellulose nanocrystals (CNCs) onto carbon fibers, constructing hierarchical interfacial connections between the fibers and the epoxy matrix and significantly improving the interfacial bonding strength. As a result, the tensile strength of the epoxy composites increased from 519 MPa to nearly 900 MPa, which provides a potential approach for significantly improving the mechanical performance of composites.

## 1. Introduction

High-performance polymeric composites, represented by fiber-reinforced resin matrix composites [1], demonstrate superior comprehensive performance [2,3] and are typically used in aerospace and other fields [4]. In recent years, several novel fiber reinforcements, such as carbon fibers [5], aramid fibers [6], and quartz fibers [7], have been developed and contributed to the development of new generations of composites [8], which have gained extensive attention in engineering fields [9,10]. Interfaces between fiber reinforcement and the resin matrix are important for determining the comprehensive performance of composites [11,12]. Interfaces undertake the tasks of transferring loads from the resin matrix to the fiber reinforcement [13] and delay the propagation of microcracks [14,15,16]. Therefore, the design and construction of a good interface have always been topical issues in composite fields [17,18].

Carbon fibers (CFs) have high specific strengths [19], high specific moduli [20], low thermal expansion coefficients, high temperature resistances, and corrosion resistances [21,22]. However, the modulus mismatch between the carbon fiber and the matrix can result in a concentration of stresses [23]. Moreover, smooth and chemically inert surfaces cause unsatisfactory resin wettability and ultimately lead to poor interfacial connections [24]. In addition, the slightly negative thermal expansion coefficient of the carbon fibers also results in a mismatch, as that of the resin is generally positive [25]. These factors make carbon fiber-reinforced resin matrix composites prone to separation between the matrix and fibers when subjected to external force [26], which is unfavorable for the development of high-performance composites [27]. Therefore, enhancing the interfacial bonding [28] between the carbon fibers and the composite material and increasing the interaction force between the two phases [29] are important for improving the uniform transfer and dispersion of the composite load between the matrix and the reinforcement [30,31] and improving the comprehensive performance of the material [32,33].

Cellulose, one of the most widely distributed natural biopolymers that demonstrates superior theoretical strength, was developed to reinforce epoxy composites. Dispersing cellulose fibers in an epoxy matrix has been proven to be an effective method to achieve mechanical enhancement [34,35]. The rigid molecule chain endows it with potential as a nanofibrous reinforcement, while the abundant hydroxyl groups provide conditions for chemical modification and interface bonding. Actually, many types of nanofillers, such as carbon nanomaterials [36,37,38], nanosmorillonite [39], silica nanoparticles [40], etc., have been applied to improve the mechanical properties of epoxy composites. Among these nanofillers, highly crystalline cellulose nanocrystals (CNCs) have emerged as prominent and noteworthy contributors. In addition to the advantages that cellulose fibers possess, highly aligned chains endow nanocrystals with a much higher theoretical tensile strength of 7.5 GPa and a modulus of 140–150 GPa. However, using cellulose nanocrystals as fillers often results in better mechanical properties but can also lead to a reduction in toughness [41,42]. 

In this study, rigid, rod-shaped, cellulose nanocrystals were covalently grafted onto amino-modified carbon fibers, which were preprepared by grafting the silane coupling agent KH792. An increase in the surface energy of carbon fibers, achieved by adding polar functional groups and changing the surface roughness, facilitated adequate resin impregnation. The constructed hierarchical interface thus tightly bonded the fiber fabrics and the epoxy matrix, contributing to the improvement in strength.

## 2. Materials and Methods

Materials. Epoxy resin (E-44) was purchased from Chengdu Shunmei Compound Materials Co., Ltd. It has a CAS number of 38891-59-7. This is an epoxy prepolymer produced by the copolymerization of bisphenol A-type epoxy resin and aliphatic epoxy resin (1,4-butanediol diglycidyl ether). Diethylphenylenediamine (E-100) was purchased from Jinan Nuoshi New Material Co., Ltd. Carbon fiber fabric (twill, 200 g/m^2^) was purchased from Chengdu Luchen New Material Technology Co., Ltd. The purchased carbon fabric is coated with a very thin layer of sizing agent containing epoxy and a silane coupling agent component, which makes it suitable for preparing epoxy composite. Cellulose nanocrystals (hydrodynamic diameter of ~92 nm, crystallinity of ~72%, prepared via the sulfuric acid hydrolysis method) were purchased from Shansi Technology. N-Aminoethyl-γ-aminopropyl ethyltrimethoxysilane (KH-792) (95 wt%) and (3-glycidylpropoxy)trimethoxyalkane (KH-560) (98 wt%) were purchased from Shanghai Macklin Biochemical Technology Co., Ltd. Ethanol (analytical grade) was purchased from Chengdu Haihong Experimental Instrument Co., Ltd.

Preparation of KH560-modified CNCs. CNC powder (0.2 g) was added to 100 g of deionized water, followed by ultrasonication (600 W, 1 h) to form a suspension. Then, KH560 with masses of 0 g, 2 g, and 4 g was added to the suspension. Note that the concentration of KH560 for modifying CNCs was determined according to previous methods [43]. The suspension was kept at 70 °C and stirred for 4 h to achieve complete reactions. The as-prepared KH560-modified CNCs were collected via centrifugation, and the precipitate was washed with ethanol and deionized water. After drying in an oven at a temperature of 60 °C, the obtained product was referred to as KH560-Y-CNCs, where Y represents the mass fraction of KH560 in the solution.

Preparation of KH792-modified CF. KH792 with varying masses of 1 g, 2 g, 4 g, 6 g, and 8 g was added into 100 g of an ethanol/water mixture solution (volume ratio of ethanol to water was 95:5). Note that the concentration of KH792 for modifying CF was determined according to previous methods [44]. A small amount of acetic acid was added to the solution to adjust the pH to 5, and the solution was stirred for 30 min at room temperature. A piece of carbon fabric with a size of 10 cm × 5 cm was then soaked in the solution for 4 h, after which the solution was placed in an oven and kept for 2 h at 120 °C. The as-prepared carbon fabrics were finally washed and dried. They were referred to as KH792-X-CF, where X represents the mass fraction of KH792 in the solution.

Preparation of CNC-modified CF and its corresponding epoxy composites. KH560-CNCs with a mass ratio of 0.2 wt% were added to deionized water and ultrasonicated for 30 min at an ultrasonic power of 600 W. KH792-CF was then added to the dispersion, which was immersed for 30 min and dried at 60 °C. The as-prepared fabrics were recorded as CNC-Y-KH560-KH792-X-CF, and in some cases, they are abbreviated as CNC-CF. The epoxy composites were made by hand lay-up. Specifically, the CNC-CFs were soaked in 100 g of defoamed epoxy resin that contained 26.4 g of diethyltoluenediamine. The CNC-CFs were then stacked in a preheated mold and subjected to hot-pressing molding to prepare the CNC-CFs-reinforced epoxy composites.

Characterization. A scanning electron microscope (SEM, GeminiSEM 300, ZEISS) was used to characterize the morphology of the carbon fibers and composites. The voltage was set to 15 kV, and the working distance was approximately 5 mm for the morphology characterizations. All the samples were mounted on metal stubs with carbon tape before imaging. For the composites, a gold coating was applied to their surface via magnetron sputtering to ensure electrical conductivity during SEM imaging. The morphology of the CNCs was characterized using transmission electron microscopy (TEM, JEM-2100F, JEOL). The CNCs’ powder was dispersed in deionized water at a concentration of 0.6 mg/mL and then dripped onto a microgrid copper mesh before characterization. The accelerating voltage for the electron beam was set to 200 kV. The infrared transmittance spectra of the samples were characterized via Fourier transform infrared spectroscopy (FT-IR, Tensor II, Bruker). The data were collected in the range of 4000–400 cm^−1^ with a spectral resolution of 2 cm^−1^. The photoelectron spectra were characterized via X-ray photoelectron spectroscopy (XPS) with a Thermo Scientific TMESCALABTM Xi + X-ray photoelectron spectrometer from Thermo Fisher. The X-ray excitation source was Al Kα, and the working vacuum level of the sample during spectrum acquisition was between 10^−7^ and 10^−9^ Torr. The surface wettability of the carbon fabrics was tested via a contact angle measuring instrument. The dispersion component, polar component, and surface energy were calculated via Equations (1) and (2). The monofilament strengths of the carbon fibers and composites were tested via an electronic universal testing machine (UTM4304X, Shenzhen Sansi Zongheng Technology Co., Ltd.). For each sample to be tested, dog-bone shaped samples, measuring 250 mm in length, 25 mm in width, and 0.5 mm in thickness, were cut using a diamond saw. The tests were conducted at a crosshead speed of 2 mm/min at ambient temperature and 50% relative humidity. Specimens were securely clamped in the machine using serrated grips to ensure uniform load application. Load and displacement data were continuously recorded until failure, from which tensile strength, apparent elastic modulus, and apparent elongation at break were calculated. Note that apparent strain is represented by the displacement distance of the strain sensor, that is, the displacement of the crosshead, which can introduce unavoidable errors in strain data and modulus. Please refer to the attachment for details. Five specimens were tested to ensure statistical reliability. Note that the as-obtained tensile strength, apparent elastic modulus and apparent elongation at break data were averaged over 5 parallel tests to ensure their reliability. The error bars that represent the standard deviations are provided accordingly.

(1) $$\gamma_{l}\left(1+\cos\theta \right)=2{\left(\gamma_{l}^{d}\gamma_{s}^{d}\right)}^{1/2}+2{\left(\gamma_{l}^{p}\gamma_{s}^{p}\right)}^{1/2}$$

(2) $$\gamma_{s}=\gamma_{s}^{d}+\gamma_{s}^{p}$$

where $\gamma_{l}$, $\gamma_{l}^{d},$ and $\gamma_{l}^{p}$ are the surface energy, chromatic dispersion component, and polar component of the immersion liquid, respectively, and where $\gamma_{s}$, $\gamma_{s}^{d},$ and $\gamma_{s}^{p}$ are the surface energy, dispersion component, and polar component of the carbon fiber, respectively.

The fiber volume fraction of carbon fiber-reinforced polymer composites was determined using the pyrolysis method. For each sample to be tested, three rectangular specimens measuring about 25 mm × 25 mm × 0.5 mm were cut using a diamond saw. Specimens were cleaned with acetone and dried at 80 °C for 2 h before testing. The initial mass of each specimen was recorded using an analytical balance (accuracy: 0.1 mg) and denoted as *m_c_*. These specimens were then placed in pre-weighed ceramic crucibles (denoted as *m*_0_) and heated in a muffle furnace to 550 °C. The temperature was maintained at 550 °C for 4 h to ensure complete decomposition of the resin matrix. After natural cooling to room temperature, the crucibles containing the remaining fibers were weighed (denoted as *m*_1_). Thus, the fiber volume fraction (*V_f_*) was calculated according to Equation (3), where *ρ_f_* and *ρ_c_* are the densities of the fiber and composite, respectively. The density of carbon fabric was 1.8 g/cm^3^ and that of composite was 1.5 g/cm^3^. All tests were performed in triplicate to ensure reproducibility and the average and standard deviation of the three specimens were reported.

(3) $$V_{f}=\frac{\frac{m_{1}-m_{0}}{\rho_{f}}}{\frac{m_{c}}{\rho_{c}}}$$

## 3. Results

**Figure 1:**
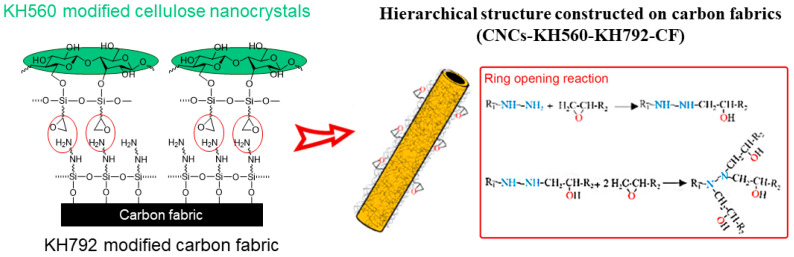
Schematics for the modification of carbon fibers by grafting CNCs with the assistance of coupling agents.

Structural modifications for smooth and inert carbon fabrics are effective strategies for improving the interfacial bonding between the fiber reinforcement and the matrix [45]. In this work, dual silane coupling agents of KH560 and KH792 were used to covalently graft CNCs onto carbon fabrics. Figure 1 shows the process. Specifically, the amino-containing silane coupling agent KH792 was hydrolyzed and grafted onto carbon fibers, and the epoxy-containing coupling agent KH560 was hydrolyzed and grafted onto CNCs. For convenience, the amino-modified carbon fibers are referred to as KH792-CF and the epoxy-modified CNCs are referred to as KH560-CNCs. Then, the amino-modified carbon fabrics reacted with the epoxy-modified CNCs and covalently bound the rigid and rod-shaped CNCs onto the carbon fabrics, which were recorded as CNCs-CF.

**Figure 2:**
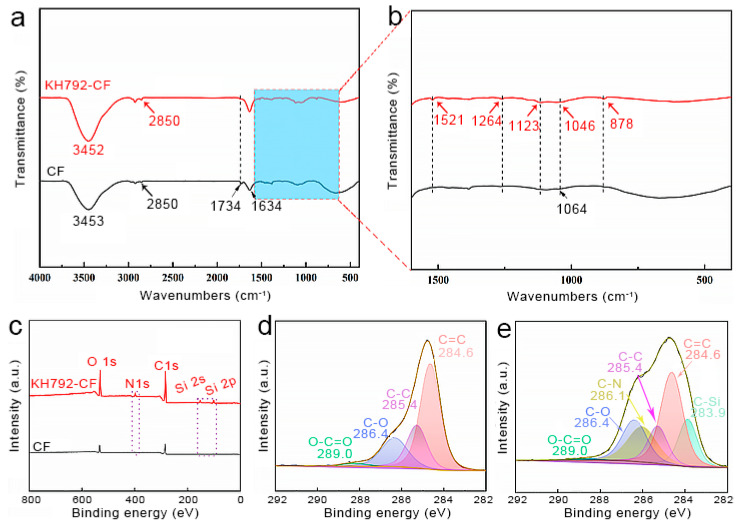
Structural characterization of KH792-modified carbon fibers before and after modification. (**a**) FT-IR spectra and (**b**) enlarged absorption peaks in the band range of 1600–400 cm^−1^. (**c**) XPS spectra. (**d**,**e**) C 1s high-resolution spectra of CF and KH792-CF.

The amino-modified carbon fibers, that is, KH792-CF, were characterized via FT-IR and XPS. The hydrolyzed KH792 contained three silanol groups. The active silanol groups can react with the hydroxyl groups present on the surface of the carbon fabric. Moreover, dehydration condensation possibly occurs between silanol groups, leading to self-condensations between KH792 molecules. The infrared spectra shown in Figure 2a demonstrate the vibrations of characteristic groups in the carbon fibers. The absorptions at approximately 1064 cm^−1^ and 1734 cm^−1^ refer to the stretching vibrations of C–O and C=O, respectively [46]. The peak located at approximately 2850 cm^−1^ represents the symmetric/antisymmetric stretching vibration of –CH_2_– [47]. The absorption peaks at 3453 cm^−1^ and 1634 cm^−1^ correspond to the stretching vibration and bending vibration of –OH, which may be due to the residual water in the carbon fibers and the –OH on the surface functional groups [48]. For KH792-CF, the enlarged spectra in Figure 2b show new peaks at 1264 cm^−1^, 1123 cm^−1,^ and 878 cm^−1^, which were attributed to the C–Si, Si–O–C, and Si–O–Si groups, respectively. These results indicate that KH792 molecules were successfully grafted onto carbon fibers and that self-condensations between KH792 molecules did occur in the as-prepared KH792-CF.

**Figure 3:**
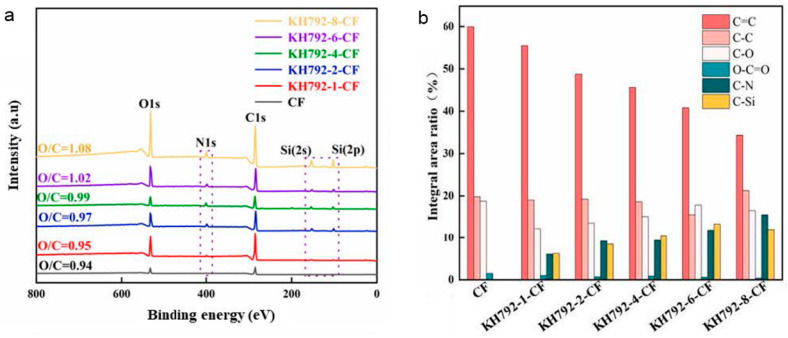
XPS results of amino-modified carbon fibers synthesized by treatment with different concentrations of KH792. (**a**) Full XPS spectrum. (**b**) Integral area of deconvoluted peaks from C 1 s high-resolution spectra.

Figure 2c shows the XPS results of the carbon fibers before and after grafting KH792. KH792-CF exhibited new peaks at 399.8 eV, 152.3 eV, and 101.2 eV, representing the electron binding energies of N 1s, Si 2p, and Si 2s, respectively [37]. These signals confirmed the existence of KH792 on the surface of the CF. The C 1s high-resolution spectrum of the CF was deconvoluted into four peaks, indicating the electron binding energy of C in O–C=O, C–O, C–C, and C=C [49]. However, for KH792-CF, peaks representing C–N and C–Si groups emerged. These results were consistent with the characterization of the FT-IR spectra, which further confirmed that KH792 was successfully grafted onto the CF. The concentration of the KH792 solution was changed to investigate its influence on the modification process. The as-prepared products were recorded as KH792-X-CF, where X represents the mass fraction of KH792 in the solution. Figure 3 shows the XPS spectra of each sample. The C 1s high-resolution spectra were deconvoluted into individual peaks, and their areas were calculated, as shown in Figure 3b. The O/C ratio of KH792-CF gradually increased from 0.94 to 1.08 as the KH792 concentration increased, and the peak areas representing the C–N, C–Si, and C–O structures also increased significantly. This suggests that an increased KH792 concentration can increase the content of amino groups grafted on CF.

CNCs were covalently modified with the epoxy-containing coupling agent KH560 prior to grafting onto KH792-CF. KH560 also formed three silanol groups after hydrolysis and condensed, with the –OH exposed on the surface of the CNCs [50]. This formed epoxy-functional cellulose nanocrystals, which were denoted as CNC-Y-KH560 (where Y represents the mass fraction of KH560 in the solution). The TEM images shown in Figure S1 reveal that the nanorod-like CNCs exhibited few changes in morphology after covalent modification.

**Figure 4:**
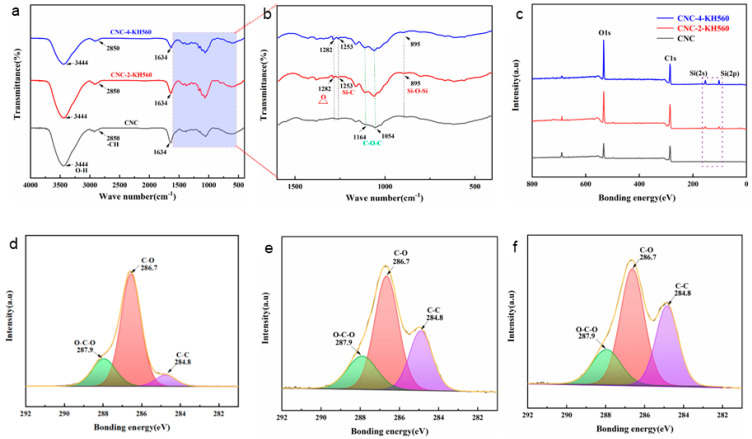
Structural characterization of KH560-grafted cellulose nanocrystals. (**a**) FT-IR spectra and (**b**) enlarged absorption peaks in a band range of 1600–400 cm^−1^. (**c**) XPS spectra. (**d**–**f**) C 1s high-resolution spectra of CNCs, CNCs-2-KH560, and CNCs-4-KH560.

Figure 4 shows the FT-IR and XPS spectra of the KH560-modified CNCs. As shown in Figure 4a,b, the newly emerged infrared absorption signal at 1282 cm^−1^ represents the epoxy functional groups, whereas the absorbance peaks corresponding to Si-C and Si-O-Si exist at 1253 cm^−1^ and 895 cm^−1^, respectively [51,52]. Figure 4c presents the XPS signal, and Si was detected on the surface of the KH560-modified CNCs. These results fully confirmed that KH560 was successfully grafted onto the surface of the CNCs. The C 1s high-resolution spectra of these samples were deconvoluted into three peaks representing the electron binding energies of C in O–C–C, C–O, and C–C [53]. We found that the proportion of C–C groups increased significantly with increasing concentration of KH560 solution and that the proportion of C–O groups decreased. These findings confirm that KH560 was grafted onto the surface of CNCs through dehydration condensation.

**Figure 5:**
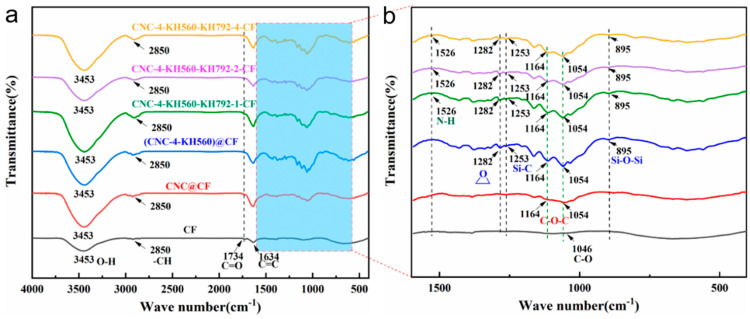
FT-IR spectra of carbon fibers grafted with CNCs. (**a**) FT-IR spectra and (**b**) enlarged absorption peaks in the band range of 1600–400 cm^−1^. CNC@CF and (CNC-4-KH560)@CF refer to the bare carbon fabrics that were loaded with CNCs and CNC-4-KH560 nanorods via solution blending. CNC-Y-KH560-KH792-X-CF refers to the CNCs-modified carbon fabrics prepared by covalently grafting with dual silane coupling agents. X represents the mass concentration of KH792 for modifying carbon fabrics and Y represents the mass concentration of KH560 for modifying CNCs.

KH560-modified CNCs were further covalently grafted onto KH792-modified CFs. This was achieved by initiating the ring opening of the epoxy groups grafted on the CNCs via the amino groups on KH792-CF. The CNCs and CNCs-4-KH560 agglomerated on the bare CFs, as shown in Figure S2b,c. However, for the amino-modified carbon fabrics, CNCs-4-KH560 were uniformly distributed on the fiber surface and finally formed a coating (Figure S2d–f). Figure 5 shows the FT-IR spectra of the CNC-grafted carbon fabrics. With an increasing degree of amination modification of the carbon fabrics, the relative intensity of the peak at 1282 cm^−1^, which represented the antisymmetric stretching vibration of epoxy groups on KH560-CNCs, decreased. This confirms that KH560-CNCs were grafted to the surface of KH792-CF through the epoxy ring-opening reaction. However, a faint peak could still be observed in the product, indicating that a small number of epoxy groups remained in the product. This means that some KH560-CNCs were indirectly fixed on the CF surfaces by dehydration condensation between silanol groups rather than the epoxy ring-opening reaction. Therefore, when many KH560-CNCs were attracted from the surface of the carbon fiber, self-polymerization between silanol groups caused agglomeration of the CNC nanorods. Moreover, a peak at 1526 cm^−1^, which represented the bending vibration of N–H bonds, was observed, indicating the existence of amino groups.

**Figure 6:**
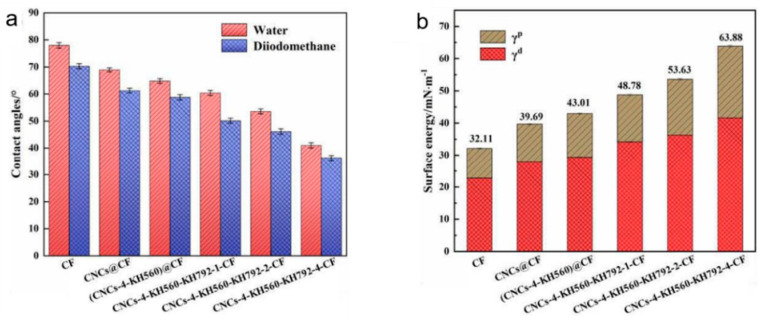
Wettability of CNCs-modified carbon fibers. (**a**) Contact angle and (**b**) surface energy.

The surface energy of carbon fabrics is a key factor affecting resin impregnation, which is important for ensuring the interfacial bonding strength. The contact angles of the CNC-modified CF with water and diiodomethane were tested, and the surface energy of the fabric was calculated via Equations (1) and (2). The results are shown in Figure 6. For comparison, the contact angles and surface energy of KH792-CF are presented in Figure S3. The surface energy of the bare CF was 32.11 mM/m, and it gradually increased to 63.88 mM/m after being modified with dual silane coupling agents and CNCs. In general, the dispersion component is related to the surface roughness, whereas the polar component is related mainly to the contents of polar groups exposed on surfaces [54]. The grafting of CNCs with KH792 and KH560 simultaneously increased the polar component and the dispersion component of the CF. The former was due to the large number of polar groups on the surface of the CNCs, and the latter was due to the grafting of cellulose nanocrystals, which increased the surface roughness of the carbon fabrics.

Since CNCs were covalently grafted onto the surface of carbon fabrics with the assistance of long-chain molecules comprising KH560 and KH792, a hierarchical surface was constructed. Owing to the aforementioned surface energy, which aided in adequate resin impregnation, rigid, rod-shaped CNCs were expected to embed into the resin matrix, achieving mechanical interlocking. Moreover, the residual amine and epoxy groups can participate in the curing of epoxy resin, and the abundant hydroxyl groups exposed on CNC surfaces can also form hydrogen bonds within the epoxy networks. All these factors contributed to improving the interfacial binding strength and enhancing the mechanical strength of the composites. On the basis of this design concept, a series of fiber-reinforced epoxy composites were prepared with bare CF, amino-modified CF (KH792-X-CF), and CNC-modified CF (CNC-KH560-KH792-X-CF) as continuous fiber reinforcement. Note that the fiber volume fractions for these representative samples were almost identical according to the experimental test result shown in Table S1. The mechanical strength of the samples was tested, and the resulting stress–strain curves are shown in Figure S4. The tensile strength of these samples is presented in Table 1. The tensile strength of the composites reinforced with bare CF were 519 MPa. This result is consistent with the mechanical property test results of carbon fiber-reinforced epoxy composites reported in the literature, indicating that the molding process for the composite material is reasonable [55,56]. For the composites reinforced with KH792-CF, the tensile strength increased to 583 MPa. This benefited from the improved resin impregnation caused by the increased surface energy (Figure S2). In addition, residual amine groups on the surface of KH792-CF can participate in the curing process of the epoxy resin, thus improving the interfacial bonding strength. After the further grafting of CNCs onto CF, the maximum tensile strength of the as-prepared epoxy composites reached nearly 900 MPa, which was 70% greater than that of the bare CF-reinforced composites. To confirm that the enhancement in mechanical properties was statistically significant, we performed a *t*-test for significance analysis. The epoxy composites reinforced with CF, KH792-2-CF, and CNCs-4-KH560-KH792-2-CF were used as representatives for performing the significance analysis, and the results are presented in Table S2. Since the p value were much lower than 0.05, we concluded that the surface modification of carbon fibers, particularly the grafting of CNCs, indeed led to a significant improvement in the overall mechanical properties of the composites. Table S3 summarizes the mechanical performance of the epoxy composites reinforced with various nanofillers, such as carbon nanotubes, montmorillonite, graphene, and graphene oxide [44,57,58,59,60,61,62]. As a comparison, the mechanical properties of our samples improved by up to 70%, higher than those reported works. 

**Figure 7:**
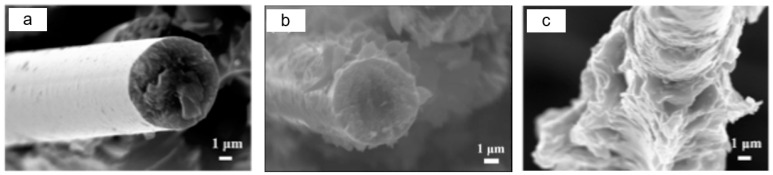
SEM images of tensile cross-sections of the carbon fiber-reinforced epoxy composites before and after modification. The fiber reinforcements are (**a**) carbon fiber (CF), (**b**) KH792-modified carbon fiber (KH792-CF), and (**c**) CNC-KH560-grafted aminated carbon fibers (CNCs-KH560-KH792-CF).

**Figure 8:**
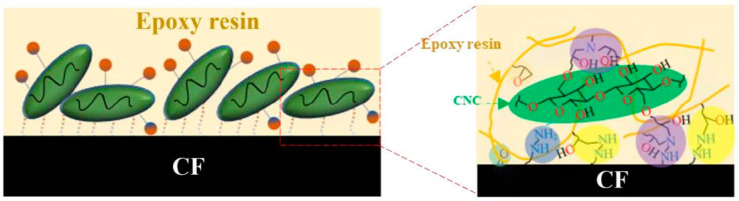
Schematic illustration of the hierarchical interfaces between a CF and an epoxy matrix for improving the mechanical performance of composites.

To analyze the interfacial bonding between the epoxy matrix and the CNC-CF reinforcements, SEM images of the fibers pulled out from the matrix are shown in Figure 7. For unmodified CFs, the extracted fibers were bare and smooth with little resin adhesion (Figure 7a). This indicated that the interfacial bonding strength between the carbon fibers and the resin matrix was weak. After modification with KH792, resin debris appeared on the surface of the pulled-out carbon fibers (Figure 7b), suggesting that an increased interfacial bonding force formed between KH792-CF and the epoxy matrix. For the CNC-CF reinforcements, the surface of the pulled-out fibers was completely covered by the resin matrix, and the resin surface exhibited a zigzag fracture morphology (Figure 7c). This means that the interfacial bonding strength between the fibers and the matrix was greater than the strength of the resin matrix; thus, tensile failure occurred inside the resin matrix rather than at the interface region. A key enhancement mechanism is that the rigid, rod-shaped CNCs covalently grafted on the CF were embedded in the epoxy matrix and formed an anchor, which significantly improved the interfacial bonding strength. Moreover, the residual amnio groups that appeared on the surface of the CNCs-CF were involved in curing the epoxy matrix, which was also conducive to improved interfacial binding. Figure 8 illustrates the hierarchical interface constructed between the CF and the epoxy matrix. The improved toughness of the composites was due primarily to the long-chain structure composed of KH560-KH792 that connected the CF and CNCs, which buffered the stress transfer process around the interfaces. The CNC layers also served as buffer layers for improving the modulus matching between the fiber reinforcement and the matrix. As a result, the mechanical performances of the CNC-CFs-reinforced epoxy composites significantly increased. 

## 4. Conclusions

CNCs were covalently grafted onto the surface of carbon fabrics with the assistance of long-chain molecules comprising KH560 and KH792. These hierarchical surfaces significantly improved the interfacial bonding strength between the CF and the epoxy matrix through mechanical anchoring, covalent bonding, and improved resin impregnation. Compared with that of unmodified CFs, the mechanical performance of the as-prepared composites was remarkably improved. The tensile strength reached nearly 900 MPa, demonstrating effective enhancement by constructing a hierarchical interface using CNCs and dual silane coupling agents. This work seems to be a feasible method to significantly improve the strength of composites.

## Figures and Tables

**Table 1:** Mechanical strength of epoxy composites prepared with KH792-modified carbon fibers (KH792-X-CF) and CNCs-modified carbon fibers (CNCs-KH560-KH792-X-CF) as reinforcements.

Samples	Tensile Strength (MPa)
CF	519 ± 20
KH792-1-CF	541 ± 24
KH792-2-CF	543 ± 8
KH792-4-CF	583 ± 10
CNCs@CF	551 ± 32
(CNCs-4-KH560)@CF	684 ± 21
CNCs-4-KH560-KH792-1-CF	734 ± 28
CNCs-4-KH560-KH792-2-CF	881 ± 22
CNCs-4-KH560-KH792-4-CF	736 ± 26

## Data Availability

The original contributions presented in the study are included in the article and Supplementary Material; further inquiries can be directed to the corresponding authors.

## Supplementary Materials

The following supporting information can be downloaded at https://www.mdpi.com/article/10.3390/nano14181537/s1: Figure S1: TEM morphology characterization of the KH560 graft-modified cellulose nanocrystals; Figure S2: SEM morphology characterization of cellular nanocrystal-grafted modified carbon fibers; Figure S3: Wettability of amino-modified carbon fibers synthesized by treating with different concentrations of KH792; Figure S4: Stress–strain curves of epoxy composites prepared with KH792-modified carbon fibers (KH792-X-CF) and cellular nanocrystalline-grafted modified carbon fibers (CNCs-KH560-KH792-X-CF) as the reinforcement. Table S1: The fiber volume fraction for different composite samples obtained through ablation testing. Table S2: Significance analysis based on *t*-test results for the mechanical performances of the epoxy resin composites reinforced with CF, KH792-2-CF, and CNCs-4-KH560-KH792-2-CF. Table S3: Comparison of mechanical properties of epoxy composites reinforced with different nanofillers. Table S4: Apparent elongation at break of epoxy composites prepared with KH792-modified carbon fibers (KH792-X-CF) and CNCs-modified carbon fibers (CNCs-KH560-KH792-X-CF) as reinforcements.
