# Supplementary material for: Hierarchical Interfacial Construction by Grafting Cellulose Nanocrystals onto Carbon Fiber for Improving the Mechanical Performance of Epoxy Composites
Source: Nanomaterials (Basel). 2024 Sep 22;14(18):1537. doi: 10.3390/nano14181537 (PMC11434848; doi:10.3390/nano14181537)
Supplement: Supplementary file 1 [file nanomaterials-14-01537-s001.zip › nanomaterials-3182369-supplementary.pdf]

Supplementary

Hierarchical Interfacial Construction by Grafting Cellulose Nanocrystals onto Carbon Fiber for Improving the Mechanical Performance of Epoxy Composites

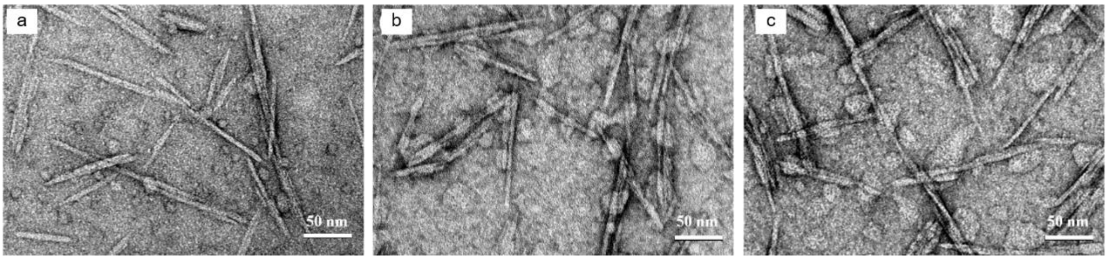

Figure S1. TEM morphology characterization of the KH560 graft-modified cellulose nanocrystals. (a) cellulose nanocrystals and (b, c) cellulose nanocrystals obtained by grafting modification with 2 wt% and 4 wt% KH560 solutions.

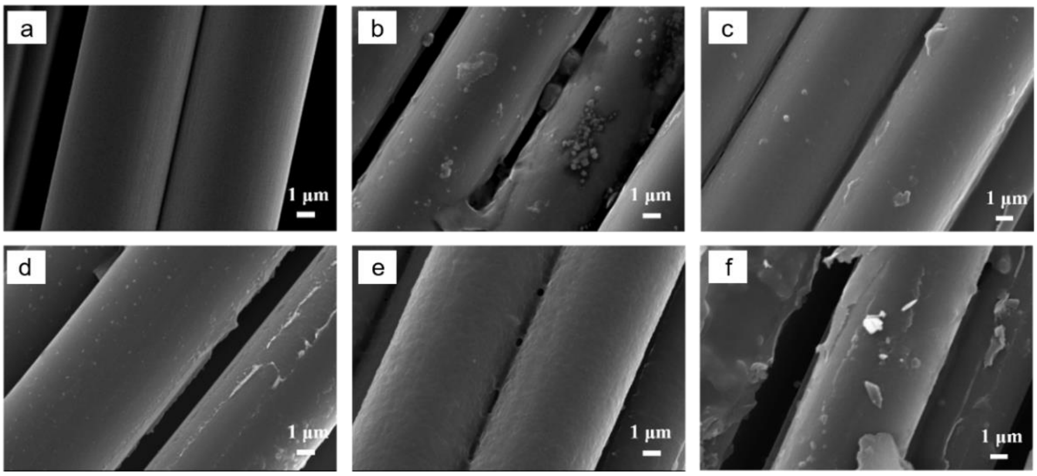

Figure S2. SEM morphology characterization of cellular nanocrystal-grafted modified carbon fibers. (a) carbon fiber (CF), (b) carbon fiber loaded with cellular nanocrystals (CNCs@CF), (c) KH560-grafted cellular nanocrystals (CNCs-4-KH560)@CF, and (d) CNCs-KH560-grafted carbon fiber (KH792-CF), denoted as CNCs-KH560-KH792-X-CF, where X represents the concentration of KH792 used in the amination modification of carbon fibers, which was 1 wt%, 2 wt% and 4 wt%, respectively.

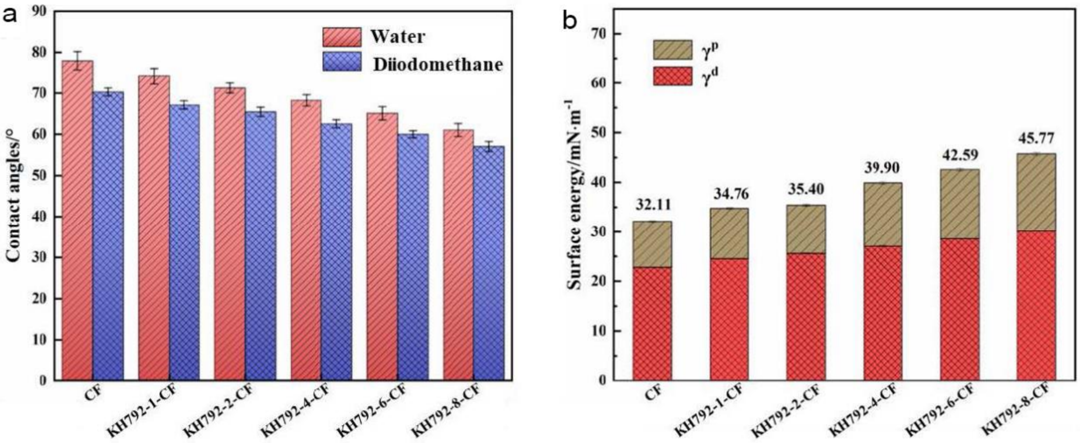

Figure S3. Wettability of amino-modified carbon fibers synthesized by treating with different concentrations of KH792. (a) Contact angle and (b) surface energy.

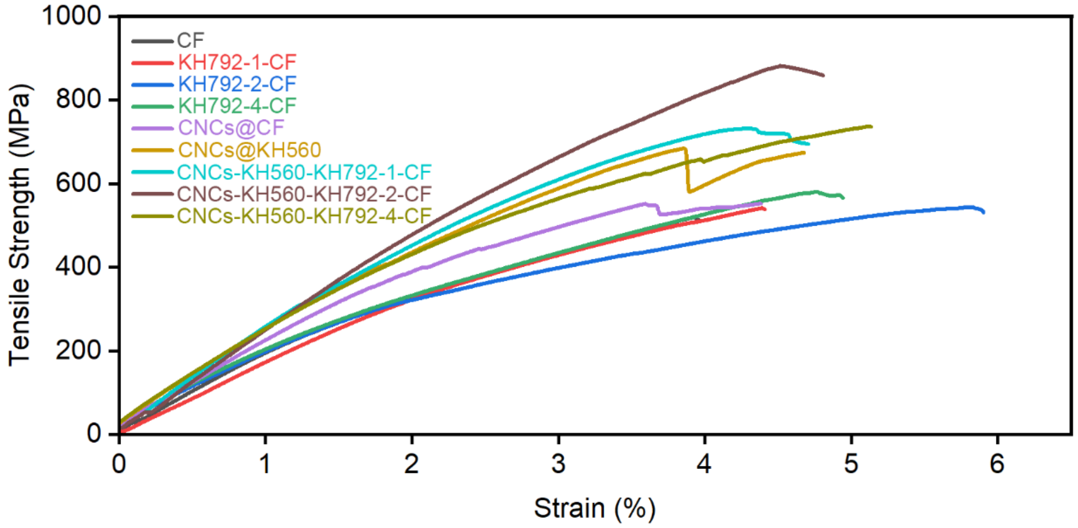

Figure S4. Stress–strain curves of epoxy composites prepared with KH792-modified carbon fibers (KH792-X-CF) and cellular nanocrystalline grafted modified carbon fibers (CNCs-KH560-KH792-X-CF) as the reinforcement.

Table S1. The fiber volume fraction for different composite samples obtained through ablation testing.

Samples	Volume fraction of carbon fibers (%)
CF reinforced composites	52.88±2.71
KH792-2-CF reinforced composites	53.37±1.79
CNCs-4-KH560-KH792-2-CF reinforced composites	52.44±2.57

Table S2. Significance analysis based on t-test results for the mechanical performances of the epoxy resin composites reinforced with CF, KH792-2-CF, and CNCs-4-KH560-KH792-2-CF.

Property	Sample comparison	t value	p value	Significant difference
Tensile Strength	CF vs KH792-2-CF	-2.5979	3.15×10^{-2}	Yes
	CF vs CNCs-4-KH560-KH792-2-CF	-29.1504	2.31×10^{-9}	Yes
	KH792-2-CF vs CNCs-4-KH560-KH792-2-CF	-36.4743	3.82×10^{-10}	Yes

Table S3. Comparison of Mechanical Properties of Epoxy Composites Reinforced with different nanofillers.

Nanofillers		Mechanical properties of composites			Ref.
Type	Contents (wt%)	Tensile strength (MPa)	Tensile modulus (GPa)	Elongation at break (%)	
Functional CNTs	0.0	681	62	-	57
	0.5	801	77	-	
CNTs	0	682	58.8	1.20	58
	0.3	741	55.7	1.32	
KH560-CNTs	0	519	12	3.9	44
	4	633	17	5.6	
Graphene nanoplates	0	852.5	22.4	3.8	59
	0.2	921.25	23.6	3.9	
Functionalized graphene	0	662.69	17.2	3.85	60
	0.5	837.30	25.4	3.3	
Graphene oxide	0	436.77	35.14	1.99	61
	2	672.08	43.40	2.25	
Montmorillonite nanoclay	0	618.18	42.80	1.87	62
	3	730.29	45.50	1.87	
CNCs (grafted with dual silane coupling agents)	0	519.29	12.67	3.74	This work
	0.2	881.21	27.59	4.63	

Table S4. Apparent elongation at break of epoxy composites prepared with KH792-modified carbon fibers (KH792-X-CF) and CNCs modified carbon fibers (CNCs-KH560-KH792-X-CF) as reinforcements.

Samples	Apparent elongation at break (%)*
CF	3.74±0.42
KH792-1-CF	4.40±0.31
KH792-2-CF	5.85±0.47
KH792-4-CF	4.77±0.57
CNCs@CF	3.59±0.29
(CNCs-4-KH560)@CF	3.87±0.44

CNCs-4-KH560-KH792-1-CF	4.33±0.30
CNCs-4-KH560-KH792-2-CF	4.63±0.41
CNCs-4-KH560-KH792-4-CF	5.16±0.43

* The apparent strain of composites and thence the apparent elongation at break was calculated according to the displacement data of crosshead. This can cause unavoidable errors and always lead to an overestimation of elongation at break.
